# Terahertz radiation generation from metallic electronic structure manipulated by inhomogeneous DC-fields

**DOI:** 10.1038/s41598-021-85619-2

**Published:** 2021-03-23

**Authors:** H. Lin, C. P. Liu

**Affiliations:** State Key Laboratory of High Field Laser Physics, Shanghai Institute of Optics and Fine Mechanics, P. O. Box 800-211, Shanghai, 201800 China

**Keywords:** Optics and photonics, Physics

## Abstract

We propose a feasible, high-efficiency scheme of primary terahertz (THz) radiation source through manipulating electronic structure (ES) of a metallic film by targeted-designed DC-fields configuration. The DC magnetic field is designed to be of a spatially inhomogeneous strength profile, and its direction is designed to be normal to the film, and the direction of the DC electric field is parallel to the film. Strict quantum theory and numerical results indicate that the ES under such a field configuration will change from a 3*D* Fermi sphere into a highly-degenerate structure whose density-of-state curve has pseudogap near Fermi surface. Wavefunctions’ shapes in this new ES are space-asymmetric, and the width of pseudogap near Fermi surface, as well as magnitudes of transition matrix element, can be handily controlled by adjusting parameter values of DC fields. Under available parameter values, the width of the pseudogap can be at milli-electron-volt level (corresponding to THz radiation frequency), and the magnitude of oscillating dipole can be at $10^{-9} C*m$-level. In room-temperature environment, phonon in metal can pump the ES to achieve population inversion.

## Introduction

The consensus that electronic structure (ES) of matter determines its optical property guides people to tailor/design suitable ES for achieving various optical applications. Compared with those secondary radiation sources^1–22^, primary radiation sources based on suitable ES are more efficient^23–43^. Here, the phrase “secondary radiation sources” refers to that radiation generation arises from nonlinear optics effect or nonlinear interaction between driving radiation beam (at other wavelength) and driven emitter materials. The phrase “primary radiation sources” refers to that the driving is not radiation or electromagnetic wave. Impurity doping, alloying, growing/synthesizing various heterostructures such as semiconductor quantum well and superlattice are popular methods of preparing novel ESs^23–43^. These methods can be categorized into chemical manipulation of ES, which refers to change matter’s chemical constituent. Clearly, the chemical manipulation can lead to “permanent” change in the ES of matter.

In contrast, fields manipulation is to modify the ES having a “volatile” change, which refers to the ES recovering to its primitive form when removing the manipulating fields. For example, De Hass–Van Alphen effect is an example of DC-(magnetic)-fields-manipulation of metal’s ES, and field-effect-transistor (FET) is an example of DC-(electric)-fields-manipulation of semiconductor carriers’ ES^44,45^. Such a “volatile” change is more appropriate for many applications because of its simplicity, efficiency and economy. The fields-manipulation is more flexible in achieving tunable solid-state radiation source because it does not need to change materials for achieving another center-frequency output.

Obvious application prospect of radiations at terahertz (THz) band^46^ promotes intensive investigations on achieving THz primary radiation sources. Scarcity of materials with ES characterized by THz-level gap leads to so-called “THz gap”^47^. Main limit of above-mentioned band-structure-engineering^23–43^ is that the designed ES comprises too many levels which often form narrow bands or so-called mini-bands. In room-temperature environment, radiation generation from such an ES in which two mini-bands spaced by a meV-level gap will have a too broad spectrum undesirable for many applications. Moreover, the dipole matrix element in such an ES is of finite enhancement than those in atomic and molecular ES.

The advantage of the fields-manipulating metallic ES over band-structure-engineering of semiconductor carriers^23–43^ is that larger charge density, as well as larger dipole magnitude, can be achieved in the former. Usually, the density of free electrons in a metal is at $10^{22\sim 23}\,{\text {cm}}^{-3}$-level, higher several order of magnitude than that of carriers in semiconductor (about $10^{10}\,\mathrm{{cm}}^{-3}$). Therefore, manipulating free electrons in a metal is more hopeful to achieve higher output power than manipulating carriers in a semiconductor.

In this work, for more practical purpose, we investigate manipulating the ES of a metallic film by a special DC-fields configuration in which the magnetic field is space-inhomogeneous. It is well-known that a homogeneous DC magnetic field $B_{0}$ can lead to an ES in which the general form of 3*D* wavefunctions is a product of a 2*D* plane-wave (in terms of $\left( z,x\right)$) and a 1*D* harmonic-oscillator wavefunction (in terms of *y*). As shown latter, orthogonality among 1*D* harmonic-oscillator wavefunctions enables the dipole matrix elements for the transition between two Landau levels in such an ES to be confined at 0 and hence prohibits dipole radiation generation from the transition between two Landau levels. In contrast, targeted using inhomogeneous $B_{0}e_{z}\sim x$ or *y* can lead to an ES whose 3*D* wavefunctions have 2*D* localized parts losing orthogonality. In such an ES, the above-mentioned prohibition on dipole radiation generation can be removed, and hence a practical radiation source is possible.

## Theory and method

### Electronic structure manipulated by homogeneous DC-fields, revisiting De Hass–Van Alphen effect

According to many textbooks^44,45^, free electrons under homogeneous DC magnetic field can be described by a class of 3*D* eigenstate wavefunctions whose general form is a product of a 2*D* plane-wave $exp\left( ik_{z}z+ik_{x}x\right)$ and a 1*D* harmonic-oscillator wavefunction $\psi ^{1D}_{n}\left( y\right)$. Here, if 3*D* eigenstate wavefunctions of a quantum system are of a general form which is a product of a 1*D* extended-state wavefunction and a 2*D* localized-state one, i.e. $\psi ^{3D}_{k_{z},n}(x,y,z)=exp(ik_{z}z)\psi ^{2D}_{n}(x,y)$, it is not certain for these 3*D* wavefunctions being orthogonal mutually. For example, if the 2*D* wavefunctions $\psi ^{2D}_{n}(x,y)$ are governed by a 2*D* Hamiltonian operator as follows:

1 $$\begin{aligned} E_{n}\psi ^{2D}_{n}=H^{2D}\psi ^{2D}_{n}=\left[ -\alpha \left( \partial _{xx}+\partial _{yy}\right) +\beta _{x} \partial _{x}+\beta _{y} \partial _{y} +V\left( x,y\right) \right] \psi ^{2D}_{n}, \end{aligned}$$

where $\alpha$, $\beta _{x}$ and $\beta _{y}$ are constants and the “potential-energy” function *V* does not contain differential operators such as $\partial _{x}$, it is easy to prove that orthogonality exists among them. It is well-known that orthogonality exists among harmonic-oscillator wavefunctions, and same conclusion is also hold for plane-waves.

In such a ES, dipole matrix elements $\int \left[ \psi ^{3D}_{k_{z},n}\right] ^{*}x,y,z\left[ \psi ^{3D}_{k_{z}+q,m}\right] dxdydz$ to be confined at 0 if $q\ne 0$ and $m\ne n$. For generating a photon with momentum $\hbar q$ and energy $\hbar qc$, momentum conservation and energy conservation demand two levels having a difference in their wavevectors. Thus, $\int \left[ \psi ^{3D}_{k_{z},n}\right] ^{*}x,y\left[ \psi ^{3D}_{k_{z}+q,m}\right] dxdydz$ are $=0$ because of orthogonality among plane-waves, and $\int \left[ \psi ^{3D}_{k_{z},n}\right] ^{*}z\left[ \psi ^{3D}_{k_{z}+q,m}\right] dxdydz$ is $=0$ because of orthogonality among harmonic-oscillator wavefunctions. In short, if 2*D*, as well as 1*D*, localized-state wavefunctions have orthogonality, these dipole matrix elements are 0, which prohibits dipole radiation generation.

In contrast, 2*D* localized-state wavefunctions can loose orthogonality for 2*D* Hamiltonian operator in suitable forms. For example, if the 2*D* Hamiltonian operator reads

2 $$\begin{aligned} H^{2D}=\left[ -\alpha \left( \partial _{xx}+\partial _{yy}\right) +f_{x} \partial _{x}+f_{y} \partial _{y} +V\left( x,y\right) \right] , \end{aligned}$$

where $f_{x}$ and $f_{y}$ are functions of variables $\left( x,y\right)$ rather than two constants, its eigenstate 2*D* wavefunctions can be not orthogonal mutually. Once 2*D* localized-state wavefunctions loose orthogonality, these dipole matrix elements, such as $\int \left[ \psi ^{3D}_{k_{z},n}\right] ^{*}z\left[ \psi ^{3D}_{k_{z}+q,m}\right] dxdydz$, can be $\sim q^{-2}\ne 0$ for cases with $q\ne 0$ and $m\ne n$ in which $\int \left[ \psi ^{3D}_{k_{z},n}\right] ^{*}x,y\left[ \psi ^{3D}_{k_{z}+q,m}\right] dxdydz=0$ still exist. This removes a prohibition on dipole radiation generation.

### Electronic structure manipulated by inhomogeneous DC-fields configuration

Above discussion enlightens us to design/tailor the Hamiltonian governing a quantum system. Hamiltonian in following form has a 2*D*-part meeting the general form Eq. (2)

3 $$\begin{aligned} H^{3D}=\frac{1}{2m}\left[ p_{x}^{2}+\left( p_{y}-eB_{0}\beta *yx\right) ^{2}+p_{z}^{2}\right] +E_{0}y, \end{aligned}$$

where inhomogeneous DC magnetic field and its vector potential read

4 $$\begin{aligned} B=\left( 0,0,B_{z}\right) =\left( 0,0,B_{0}*\beta *y\right) ; \end{aligned}$$

5 $$\begin{aligned} A=\left( 0,A_{y},0\right) =\left( 0,B_{0}*\beta *y*x,0\right) , \end{aligned}$$

$B_{0}$, $\beta$ and $E_{0}$ are constants. Although Hall current, which is common for metal under $E\times B$ configuration, can exist (because *B* is along *z*-axis and *E* is along *y*-axis in this configuration and hence a Hall current is along *x*-axis), this Hall current represents a secondary effect and is too weak compared with controlling current in solenoid responsible for producing *B*. Usually, perturbation Hamiltonian associated with such a Hall current (which is a consequence of another stronger perturbation Hamiltonian), is taken as too weak to being added into total Hamiltonian.

**Figure 1:**
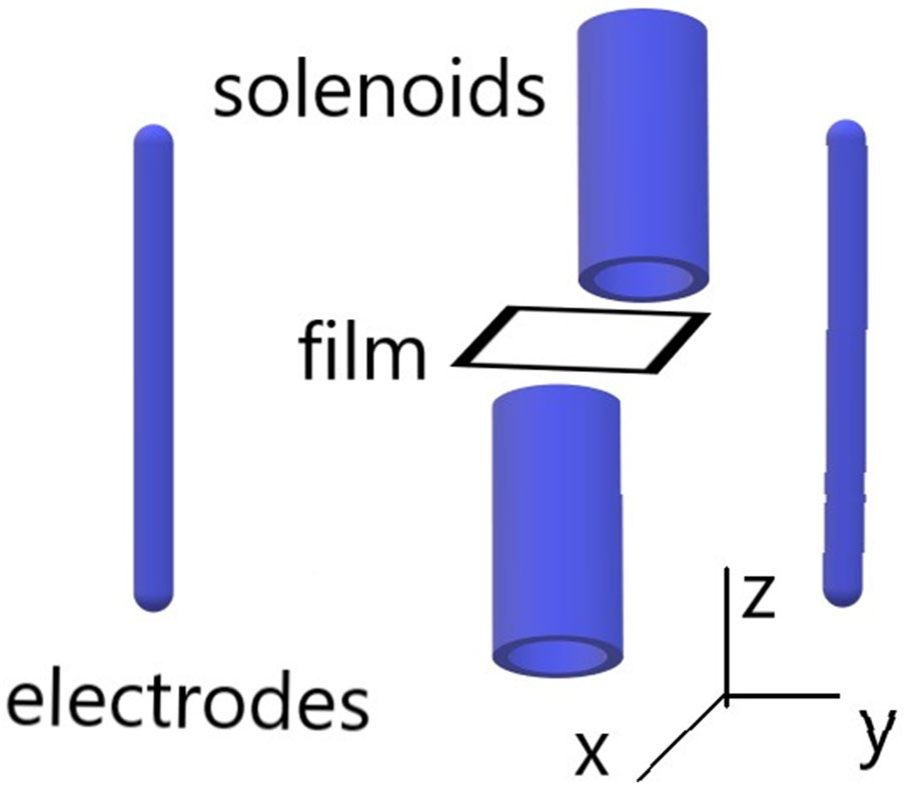
Sketch of field configuration and experimental setup. Note that two gaps might be filled with high-permittivity insulator, which can also act as an “holder” holding the film. If the “holder” is undertaken by thin dielectric substrate, suitably adjusting charge current in coils/solenoids can still warrant the metallic film to feel a magnetic slope. Note that cross-sections of solenoids are of square shape, which is not reflected in the scheme (due to the limit of plot software) and hence have to be stated clearly here.

Corresponding experimental setup is illustrated as Fig. 1. A pair of Helmholtz square-cross-section coils is on purpose mis-aligned to be not co-axial and hence produces a magnetic “slope”, which refers to $B_{z}^{2}$ is the function of *y*. The metallic film is separated from two electrode plates by two gaps, which might be filled with high-permittivity insulator or not. According to detailed description elsewhere^48^, it is feasible to attain a dimensionless slope $\beta$ up to $\upmu {\text {m}}^{-1}$-level or higher. Actually, numerous theoretical investigations on quantum Hall effect (QHE) are based on above Hamiltonian *H* at $B_{0}*\beta *y \equiv constant$-case^49^.

Electronic wave-function can be solved from

6 $$\begin{aligned} i\hbar \partial _{t}\psi =H^{3D}\psi ; \end{aligned}$$

in following general form

7 $$\begin{aligned} \psi =exp\left[ ik_{z}z-i\frac{E_{k}}{\hbar }t\right] \phi \left( x,y\right) , \end{aligned}$$

where $E_{k}$ is space-independent. Dividing both sides of Eq. (6) with $\phi$, we obtain

8 $$\begin{aligned} r.h.s=\alpha *\left\{ \left[ \partial _{xx}ln\phi +\left( \partial _{x}ln \phi \right) ^{2}\right] +\left[ \partial _{y}\left( \partial _{y}ln\phi +iwxy\right) +\left( \partial _{y}ln \phi +iwxy\right) ^{2}\right] -s y\right\} +\alpha *k_{z}^{2}; \end{aligned}$$

where constants are defined as

9 $$\begin{aligned} w= & {} -\frac{eB_{0}\beta }{\hbar }=-\left[ B_{0}\right] _{Tesla}*\left[ \beta \right] _{\upmu {\text {m}}^{-1}}*\frac{1.6}{1.054}\times 10^{3} \upmu {\text {m}}^{-3}; \nonumber \\ s= & {} -2i\frac{m_{e}E_{0}}{B_{0}\hbar \beta }=-2i*\frac{9.11}{1.054}\times 10^{-3}* \left[ E_{0}\right] _{volt/meter}/\left[ B_{0}\right] _{Tesla}/\left[ \beta \right] _{\upmu {\text {m}}^{-1}};\nonumber \\ \alpha= & {} \frac{\hbar ^{2}}{2m_{e}}\frac{1}{\upmu {\text {m}}^{2}}=\frac{1.055^2}{2*0.91*1.602}\times 10^{-7}\,{\text {eV}}=1/2.88\times 10^{-7}\,{\text {eV}}. \end{aligned}$$

Here, $\left[ B_{0}\right] _{Tesla}$ is the value of $B_{0}$ in unit of *Tesla*, and other similar symbols are explained in the same manner.

By introducing new variables

10 $$\begin{aligned} y-x/s= & {} u+v; x+y/s=u-v; \end{aligned}$$

11 $$\begin{aligned} u= & {} \frac{s+1}{2s}y+\frac{s-1}{2s}x; v=\frac{s-1}{2s}y-\frac{s+1}{2s}x; \end{aligned}$$

we have

12 $$\begin{aligned} y= & {} \frac{s+1}{s^{2}+1}su+\frac{s-1}{s^{2}+1}sv; \end{aligned}$$

13 $$\begin{aligned} x= & {} \frac{s-1}{s^{2}+1}su-\frac{s+1}{s^{2}+1}sv; \end{aligned}$$

14 $$\begin{aligned} xy= & {} \frac{s^{4}-s^{2}}{\left[ s^{2}+1\right] ^{2}}\left[ u^{2}-v^{2}\right] -\frac{4s^{3}}{\left[ s^{2}+1\right] ^{2}}uv; \end{aligned}$$

15 $$\begin{aligned} \partial _{y}= & {} \frac{s+1}{2s}\partial _{u}+\frac{s-1}{2s}\partial _{v}; \end{aligned}$$

16 $$\begin{aligned} \partial _{x}= & {} \frac{s-1}{2s}\partial _{u}-\frac{s+1}{2s}\partial _{v}; \end{aligned}$$

and can verify

17 $$\begin{aligned} d_{v}u= & {} \left[ \frac{s*\left( s-1\right) }{s*s+1}d_{y}-\frac{s*\left( s+1\right) }{s*s+1}d_{x}\right] \left[ \frac{s+1}{2s}y+\frac{s-1}{2s}x\right] \nonumber \\= & {} \frac{s*\left( s-1\right) }{s*s+1}*\frac{s+1}{2s}-\frac{s*\left( s+1\right) }{s*s+1}*\frac{s-1}{2s}=0, \end{aligned}$$

which suggests independence between *u* and *v*. Thus, we re-write Eq. (8) as

18 $$\begin{aligned} R.H.S=\frac{r.h.s}{\alpha }-k_{z}^{2}=\partial _{x}M+M^{2}+\partial _{y}N+N^{2}+\eta *\left[ \partial _{x}N-\partial _{y}M\right] ; \end{aligned}$$

where $$\eta =\frac{s}{iw}$$ and

19 $$\begin{aligned} N= & {} \partial _{y}ln\phi +iwxy;M=\partial _{x}ln\phi ; \end{aligned}$$

20 $$\begin{aligned} \partial _{x}N-\partial _{y}M= & {} iwy. \end{aligned}$$

Thus, finally we have

21 $$\begin{aligned} E_{k}/\alpha -k_{z}^{2}=R.H.S=\left[ \partial _{u}\left( N+M\right) +\frac{1}{2}\left( N+M\right) ^{2}\right] +\left[ \partial _{v}\left( N-M\right) +\frac{1}{2}\left( N-M\right) ^{2}\right] ; \end{aligned}$$

We can express $N+M$ and $N-M$ as binary power series

22 $$\begin{aligned} N-M= & {} \sum _{i,j}f_{i,j}u^{i}v^{j}; \end{aligned}$$

23 $$\begin{aligned} N+M= & {} \sum _{i,j}g_{i,j}u^{i}v^{j}; \end{aligned}$$

where coefficients $f_{i,j}$ and $g_{i,j}$ are complex-valued constants. These expansion coefficients can be solved from above equations through following strict procedure: for Eqs. (19, 20), order-by-order comparing their terms proportional to constant $u^{1}$ and $v^{1}$, we can obtain 6 algebra equations. For Eq. (21), 2 algebra equations can be obtained by order-by-order comparing its terms proportional to $u^{1}$ and $v^{1}$. Moreover, $\partial _{x} Eq. (21)=0$ and $\partial _{y} Eq. (21)=0$ also lead to 2 algebra equations. Note that 2 formulas $g_{0,0}=i\left( k_{y}+k_{x}\right)$ and $f_{0,0}=i\left( k_{y}-k_{x}\right)$ can be derived from definitions Eq. (19). Thus, 12 algebra equations and formulas (see “Appendix”) can determine solutions of these these low-order expansion coefficients $g_{1,0},g_{0,1},f_{1,0},f_{0,1},f_{1,1},g_{1,1}$. In this strict procedure, $g_{0,2},g_{2,0},f_{2,0},f_{0,2}$ act as intermediate variables. Finally, $E_{k}-E_{k}^{0}$ is a function of $g_{1,0}$ and $f_{0,1}$ and hence can be known after these low-order expansion coefficients are solved according to this procedure (whose detailed formulas are presented at “Appendix” section).

According to formulas in “Appendix” Eq. (83), $E_{k}$ is still $\sim k_{z}^{2}$ but has a more complicated dependence on $k_{\parallel }^{2}\equiv k_{x}^{2}+k_{y}^{2},\theta \equiv arctan\left( \frac{k_{y}}{k_{x}}\right)$. This will lead to corresponding variation in density-of-state (DOS) curves. Moreover, all wavefunctions shape $|\psi _{k}|$ are space-asymmetric and hence correspond to dipole moment $d_{x}\equiv \int |\psi _{k}|^{2}xdxdy \ne 0\ne \int |\psi _{k}|^{2}ydxdy\equiv d_{y}$.

### Density-of-state of the ES

When external fields are absent, conduction-band of a metal corresponds to a Fermi sphere ES in 3*D* *k*-space and has a top at Fermi surface $|k|=k_{F}$ and a bottom at $|k|=0$. Usually for light metals, their $k_{F}$ are at $10^{4}\,\upmu {\text {m}}^{-1}$-level^44,45^. In the De Hass-Van Alphen effect, the ES becomes a set of 2*D* cylindric surfaces in 3*D* *k*-space and each cylindric surface corresponds to a sub-band^44,45^. Above result implies that, under inhomogeneous DC-fields configuration, the ES changes from a 3*D* Fermi sphere in which $E_{k}^{0}\sim k_{x}^{2}+k_{y}^{2}+k_{z}^{2}$ and $0\le |k|<k_{F}$ exist, to a corrugated 3*D* Fermi sphere.

**Figure 2:**
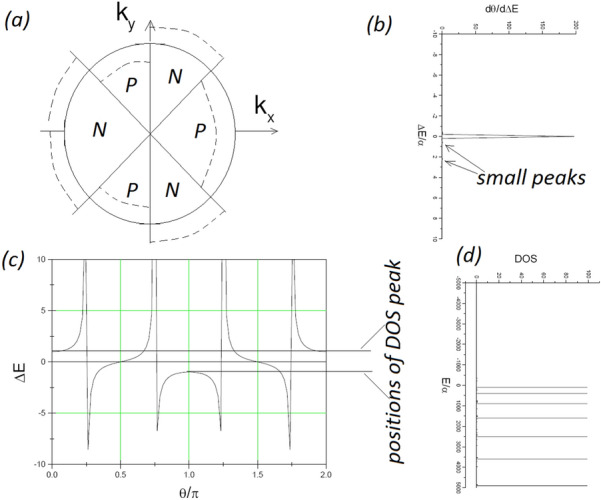
(**a**) Sketch illustrating energy shift varying over the 2*D* Fermi circle, where P (N) represents positive (negative) energy shift. The solid circle represents “unperturbed” 2*D* Fermi circle, and dashed lines represent realistic 2*D* Fermi surface. (**c**) is the dependence of the energy shift $\Delta E$ on $\theta \equiv arctan\left( \frac{k_{y}}{k_{x}}\right)$, where the definition of $\Delta E$ can be referred to Eq. (83). (**b**) is an example of DOS curve, $\sim \frac{d\theta }{d\Delta E}$, associated with the $\theta -\Delta E$ curve in (**c**). (**d**) illustrating that the superposition of DOS-curves like that in (**c**) can form DOS curve with pseudogap.

As shown in Fig. 2, each 2*D* cross-section (on the $k_{x}-k_{y}$ plane) of the 3*D* Fermi sphere can be divided into 6 segments, 3 corresponding to positive energy shift and 3 for negative. For $\theta =\frac{\pi }{4}, \frac{3\pi }{4}, \frac{5\pi }{4}, \frac{7\pi }{4}$, the energy shift $\Delta E$ can be $\pm \infty$ and correspond to nearly zero DOS, or $\frac{d\theta }{d\Delta E}\sim 0$ if $\theta =\frac{\pi }{4}, \frac{3\pi }{4}, \frac{5\pi }{4}, \frac{7\pi }{4}$.

Thus, for minimizing total energy, some regions on the $k_{x}-k_{y}$ plane, which are “unoccupied” or outside 2*D* Fermi circle, will be occupied because of negative energy shift over them, and some regions, which are “occupied” or inside 2*D* Fermi circle, will be unoccupied because of positive energy shift over them. For each $|k_{\parallel }|$-value, the dependence of the energy shift on $\theta$, according to Eq. (83), will lead to a DOS curve with pseudogap (see Fig. 2c,b) because the DOS can arrive at its maximum at two $\theta$-values: $\theta =0$ and $\theta =\pi$. If each $|k_{\parallel }|$-value has such a DOS curve with pseudogap, their superpositions, as shown in Fig. 2d, will be possible to remain a pseudogap.

**Figure 3:**
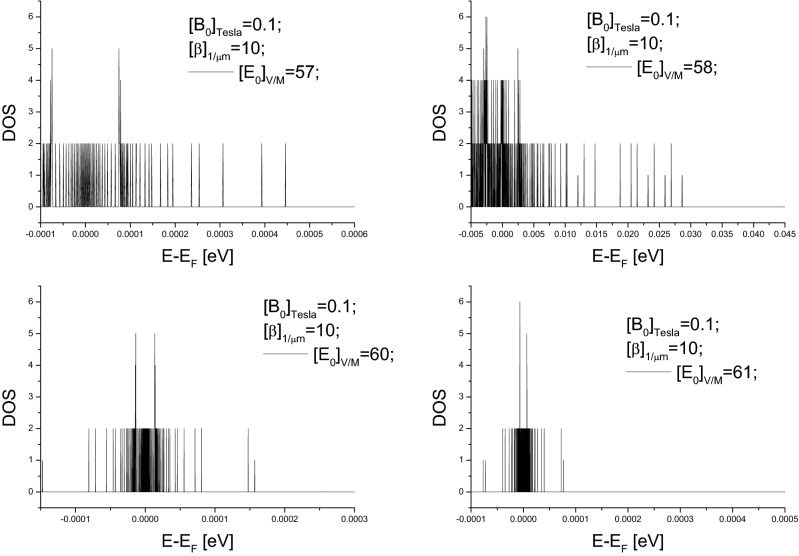
Example illustrating the dependence of the pseudogap width of DOS curve on controllable parameters.

**Figure 4:**
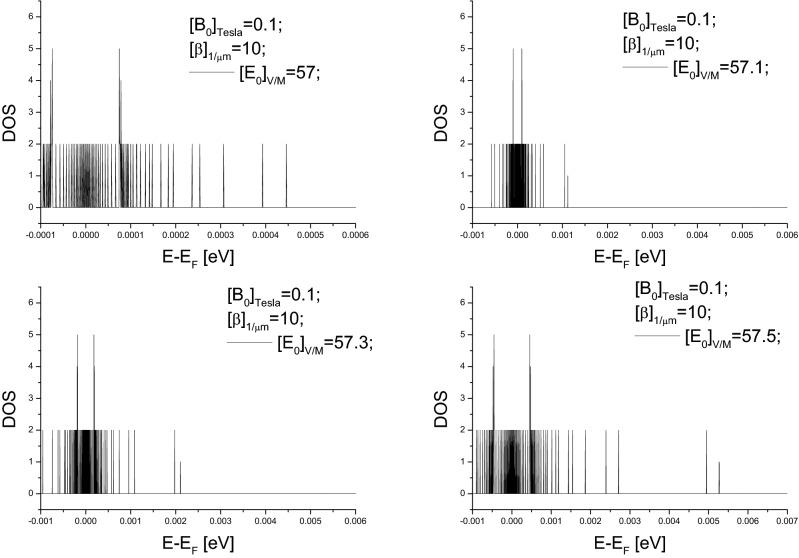
Example illustrating meV-level pseudogap width.

The pseudogap can be tuned, under available values of controllable parameters, to be at meV-level. According to Eq. (83), the fact $s^{2}<0$ (see Eq. 9) enables the width of the pseudogap at each $k_{\parallel }$-value, or $\frac{8ws^{4}}{\left[ s^{2}+1\right] ^{2}}*\frac{1}{|k_{\parallel }|}$, to be large enough. Moreover, the maximum of allowed $k_{\parallel }$-value corresponds to the narrowest among pseudogaps. Thus, as shown in Figs. 3 and  4 choosing suitable $s^{2}$-value, i.e., choosing suitable values of these controllable parameters, can lead to the superposition of these DOS curves over all allowed $k_{\parallel }$-values having a pseudogap with a desirable width. Because $1\,{\text {meV}}=10^{-3}\,{\text {eV}}=1.6 \times 10^{-19-3}$ J energy gap corresponds to a photon frequency $\nu =\frac{1.6 \times 10^{-19-3}J}{6.63 \times 10^{-34}J*S}=0.25 \times 10^{12}S^{-1}=0.25$ THz, results presented in Figs. 3 and  4 indicate that the pseudogap can be tuned from $10^{-4}$ to $10^{-2}$ eV, and hence the central frequency of generated radiations can be tuned from 25 GHz to 2.5 THz. For example, up-right panel in Fig. 3 indicates a 5 meV pseudogap and hence implies a 1.25 THz central frequency, down-right panel in Fig. 4 indicates a 2 meV pseudogap and hence implies a 0.5 THz central frequency.

**Figure 5:**
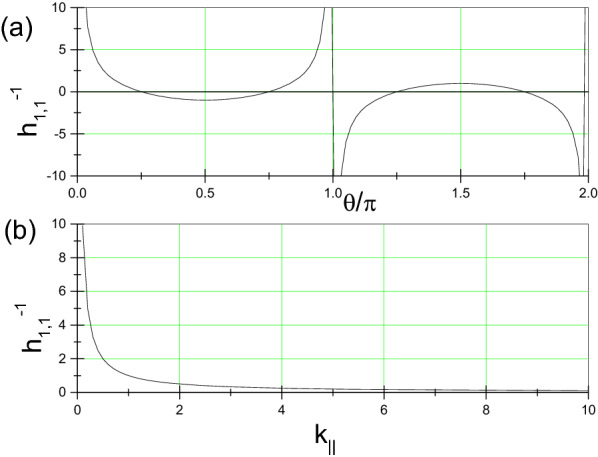
(**a**) $h_{1,1}^{-1}$ VS $\theta \equiv arctan\left( k_{y}/k_{x}\right)$ under a given $k_{\parallel }\equiv \sqrt{k_{x}^{2}+k_{y}^{2}}$ and (**b**) $h_{1,1}^{-1}$ VS $k_{\parallel }$ under a given $\theta$, where $h_{1,1}\equiv \left[ \partial _{x}\partial _{y}ln \phi \right] |_{x=0,y=0}$ and hence $h_{1,1}$ is proportional to the dipole moment $\int |\phi |^{2}xdxdy$ or $\int |\phi |^{2}ydxdy$.

Space-asymmetric wavefunction shape $|\phi _{k_{x},k_{y}}|$ implies each *k*-state corresponding to a dipole moment $d_{x,y}\equiv \int |\psi _{k}|^{2}x,ydxdy$. According to “Appendix”, the lowest order expansion terms of $Re ln\phi _{k_{x},k_{y}}$ is $\sim xy$. Thus, values of $d_{x,y}$ are dependent of a parameter $h_{1,1}\equiv \left[ \partial _{x}\partial _{y}ln \phi \right] |_{x=0,y=0}$. Larger $|h_{1,1}|$-value will correspond to larger dipole moment. As reflected by Fig. 5, larger $k_{\parallel }$-values, which correspond to states near Fermi surface, correspond to larger $|h_{1,1}|$-values.

According to familiar formula for radiation generation from quantum transition^44,45^, the radiation strength *I*, or transition matrix element, is proportional to both the product of DOS of initial state and that of final state and the product of dipole moment of initial state and that of final state:

24 $$\begin{aligned} I\left( \nu \right) \sim \sqrt{d_{E}}DOS\left( E\right) *\sqrt{d_{E+\hbar \nu }}DOS\left( E+\hbar \nu \right) . \end{aligned}$$

Note that high dipole moment and high DOS can be simultaneously achieved at some $\theta$-regimes. For example, Fig. 5a indicates that $h_{1,1}$ can be large enough at $\theta =0.5\pi$, and Fig. 2c indicates that $\theta =0.5\pi$ corresponds to a very flat segment of the $\theta - \Delta E$ curve and hence to a large DOS-value. Therefore, the corrugated ES described above, which has a tunable width of pseudogap and sufficiently high dipole moments at states near peaks around pseudogap in DOS curves, is very favorable to powerful radiation output.

The partition function of the ES, which is taken as a canonical ensemble, reads

25 $$\begin{aligned} Z=\sum _{i}g_{i}exp\left( -E_{i}/K_{B}T\right) , \end{aligned}$$

where $K_{B}$ is Boltzmann’s constant. The ground state of such an ES is to fill the complicated 3*D* body and macroscopically corresponds to a static dipole (because all $\left( k_{z},k_{\parallel },\theta \right)$-states are of “polarized” shapes).

### Parameters in laser rate equations

The new ES has a main feature: wavefunction $|\psi _{k_{x},k_{y},k_{z}}>=|\psi _{k_{x},k_{y}}\left( x,y\right) |exp\left( ik_{x}x+ik_{y}y\right) exp\left( ik_{z}z\right) exp\left( i\omega _{k}t\right)$ have a space-varying shape $|\psi _{k_{x},k_{y}}\left( x,y\right) |$. The ES of a quantum system determines its values of key parameters in rate equations of radiation generation. For example, the spontaneous emission lifetime $t_{spon,12}$ is defined as

26 $$\begin{aligned} 1/t_{spon,12}=A_{12}\equiv \frac{e^2\omega _{0}^3n^3}{3\pi c^3\hbar \varepsilon }|<\psi _{1}|r|\psi _{2}>|^2, \end{aligned}$$

where $\hbar \omega _{0}\equiv E_{2}-E_{1}>0$, and other parameters are of respective customary definitions^48^. The value of $A_{12}$ depends on not only energies of two levels, $E_{2}$ and $E_{1}$, but also space shapes of their wavefunctions, $|\psi _{2}|$ and $|\psi _{1}|$.

It is well-known that the dipole matrix element of an ES is proportional to the characteristic scale of wavefunctions of the ES, $|<\psi _{1}|r|\psi _{2}>| \equiv \xi ^{-3}\int |\psi _{1}\left( r/\xi \right) |r|\psi _{2}\left( r/\xi \right) |d^{3}r \sim \xi$. For small quantum systems such as atoms and molecules, their wavefunctions have small space extension or highly localized. For atomic wavefunctions, these exist $|\psi ^{atom}|\sim exp\left( -r/\xi \right)$ and $\xi \sim 0.053$ nm or Bohr radius (see many textbooks). As a consequence, the value of dipole matrix element of an atom $|<\psi _{1}|x,y,z|\psi _{2}>| \sim \xi$ will be about at *nm*-level and hence corresponding $t_{spon,12}$-values, for $|E_{2}-E_{1}|$ at *eV*-level, is $\sim 10^{-9}$ s (see Eq. 8.3.12 at page 168 of Ref.^50^). Similar results hold for small molecules. Moreover, in semiconductor quantum well and superlattice, wavefunctions of carriers are of larger $\xi$-value, usually tens *nm*, than atomic and molecular wavefunctions. Impurity levels also have larger $\xi$-value because of larger effective electronic mass in Hydrogen-like model on impurity. In contrast, extended-state wavefunctions in the ES of a metal is of larger $\xi$-values, as well as larger values of dipole matrix element, than those in quantum well and superlattice.

The wavefunctions’ shape also affects electron–phonon coupling constant. Electron–phonon interaction can be represented by a perturbation Hamiltonian density^45^

27 $$\begin{aligned} H^{1}_{e-ph}= & {} u\cdot \nabla V\left( r-R_{n}\right) =\sum _{n} d_{n}\cdot \nabla V\left( r-R_{n}\right) \nonumber \\= & {} A_{q}\sum _{n} cos\left( q\cdot R_{n}-\omega t\right) e\cdot \nabla V\left( r-R_{n}\right) , \end{aligned}$$

where $d_{n}=A_{q}cos\left( q\cdot R_{n}-\omega t\right)$ is the displacement of the *n*-th atom and *V* is atomic potential. Corresponding scattering probability, or electron–phonon coupling constant $g_{1,2}$, reads^45^

28 $$\begin{aligned} M_{2,1}\,= & {} g_{2,1}^{2}\equiv |<\psi _{1}|u\cdot \nabla V_{i}|\psi _{2}>|^{2} \nonumber \\= \, & {} \frac{2\pi ^{2}}{\hbar } \Bigg \{|<2|\frac{A_{q}}{2}\sum _{n} exp\left( iq\cdot R_{n}\right) e\cdot \nabla V\left( r-R_{n}\right) |1>|^{2}\delta \left[ E_{k^{'}-E_{k}-\hbar \omega }\right] \nonumber \\&+|<2|\frac{A_{q}}{2}\sum _{n} exp\left( -iq\cdot R_{n}\right) e\cdot \nabla V\left( r-R_{n}\right) |1>|^{2}\delta \left[ E_{k^{'}-E_{k}+\hbar \omega }\right] \Bigg \} \nonumber \\= \,& {} M_{1,2}; \end{aligned}$$

where phonon number $A_{q}^{2}$ can be obtained from Bose–Einstein distribution function: $g_{BE}\left( \omega _{q};T\right) \equiv \frac{1}{exp\left( \hbar \omega _{q}/K_{B}T\right) -1}$. Clearly, $M_{1,2}$ depends on phonon number $A_{q}^{2}$ and space shapes of $|\psi _{1}>$ and $|\psi _{2}>.$

Although both the electron–phonon coupling constant and the spontaneous emission lifetime are affected by the space shapes of wavefunctions, their dependencies on the shapes are different. This implies that with the ES changing (i.e., shapes of wavefunctions changing), the ratio among these coefficients in rate equations of radiation generation (see below) also change and hence so do its solutions^51,52^. Moreover, there is difference between the scattering by longitudinal-wave acoustic phonon $u_{lo}=exp(iq_{x}x+i\omega ^{lo}_{q}t)e_{x}$ and that by transverse-wave one $u_{tr}=exp(iq_{x}x+i\omega ^{tr}_{q}t)e_{y}$. In the former case, $u\cdot \nabla V \sim x*x$ while in the latter case, $u\cdot \nabla V \sim x*y$ (because ionic potential *V* is a function of $x^{2}+y^{2}+z^{2}$). Thus, the difference between two electron–phonon coupling constants is dependent on shapes of wavefunctions

29 $$\begin{aligned}&g_{2,1}^{tr}\sim \int |\psi _{1}|iqxy|\psi _{2}|dxdy; \end{aligned}$$

30 $$\begin{aligned}&g_{2,1}^{lo}\sim \int |\psi _{1}|iqx^{2}|\psi _{2}|dxdy; \end{aligned}$$

Likewise, the spontaneous emission coefficient $A_{12}$ also depends on shapes of wavefunctions in a different manner

31 $$\begin{aligned} A_{12}\sim \int |\psi _{1}|\sqrt{x^{2}+y^{2}}|\psi _{2}|dxdy. \end{aligned}$$

The rate equations of radiation generation based on 2-level read

32 $$\begin{aligned} d_{t}P_{2}= & {} P_{1}P_{2}*\sigma -A*P_{2}; \end{aligned}$$

33 $$\begin{aligned} d_{t}P_{1}= & {} -P_{1}P_{2}*\sigma ; \end{aligned}$$

where $P_{2}$ and $P_{1}$ are populations of two levels. Here, because conventional components for achieving amplified radiation, such as reflecting mirrors or resonant cavity^52^, are not used, there is no feedback usage of generated radiation and hence the population inversion is merely due to the phonon pump, i.e., not amplified. The output radiation is indeed an enhanced spontaneous emission from an inverted equilibrium population. Because no radiation is input, such an enhanced spontaneous emission can also be viewed as an infinite-fold amplification, i.e., the enhancement in spontaneous emission is viewed as infinitely amplifying 0 input radiations. Therefore, we borrow the phrase “stimulated emission”, despite not very accurate, to refers to this enhancement in spontaneous emission.

Equations (32, 33) can be derived in following way. If phonon scattering is not taken into account, populations of two levels are

34 $$\begin{aligned} P_{2}^{0}= & {} N\left( E_{2}\right) *g_{FD}\left( E_{2};T\right) ; \end{aligned}$$

35 $$\begin{aligned} P_{1}^{0}= & {} N\left( E_{1}\right) *g_{FD}\left( E_{1};T\right) , \end{aligned}$$

where $N\left( E_{2}\right)$ and $N\left( E_{1}\right)$ are DOS of two levels, respectively. During $\Delta t$ the number of electrons transiting from $E_{2}$ to $E_{1}$, due to phonon-scattering, is^45^

36 $$\begin{aligned} b=g_{FD}\left( E_{2};T\right) *N\left( E_{2}\right) *M_{2,1}*N\left( E_{1}\right) *\left( 1-g_{FD}\left( E_{1};T\right) \right) *\Delta t. \end{aligned}$$

where the factor $\left( 1-g_{FD}\left( E_{1};T\right) \right)$ represents un-occupation probability of the $E_{1}$-state and hence means that the transition is prohibited if the $E_{1}$-state has been occupied. Likewise, during $\Delta t$ the number of electrons transiting from $E_{1}$ to $E_{2}$ is^45^

37 $$\begin{aligned} a=g_{FD}\left( E_{1};T\right) *N\left( E_{1}\right) *M_{1,2}*N\left( E_{2}\right) *\left( 1-g_{FD}\left( E_{2};T\right) \right) *\Delta t. \end{aligned}$$

Therefore, after taking into account the phonon scattering, populations of two levels read

38 $$\begin{aligned} P_{2}= & {} P_{2}^{0}-b+a; \end{aligned}$$

39 $$\begin{aligned} P_{1}= & {} P_{1}^{0}+b-a, \end{aligned}$$

where

40 $$\begin{aligned} \frac{a-b}{\Delta t}= & {} N\left( E_{1}\right) *M_{1,2}*N\left( E_{2}\right) *g_{FD}\left( E_{2}\right) *g_{FD}\left( E_{1}\right) * \nonumber \\&*\left[ exp\left( E_{2}/K_{B}T\right) -exp\left( E_{1}/K_{B}T\right) \right] >0 \end{aligned}$$

Equations (38, 39) can be re-written as Eqs. (32, 33) if introducing

41 $$\begin{aligned} \sigma =M_{1,2}*\left[ exp\left( E_{2}/K_{B}T\right) -exp\left( E_{1}/K_{B}T\right) \right] \end{aligned}$$.

### Radiation generation from 3-level model and 2-level model

**Figure 6:**
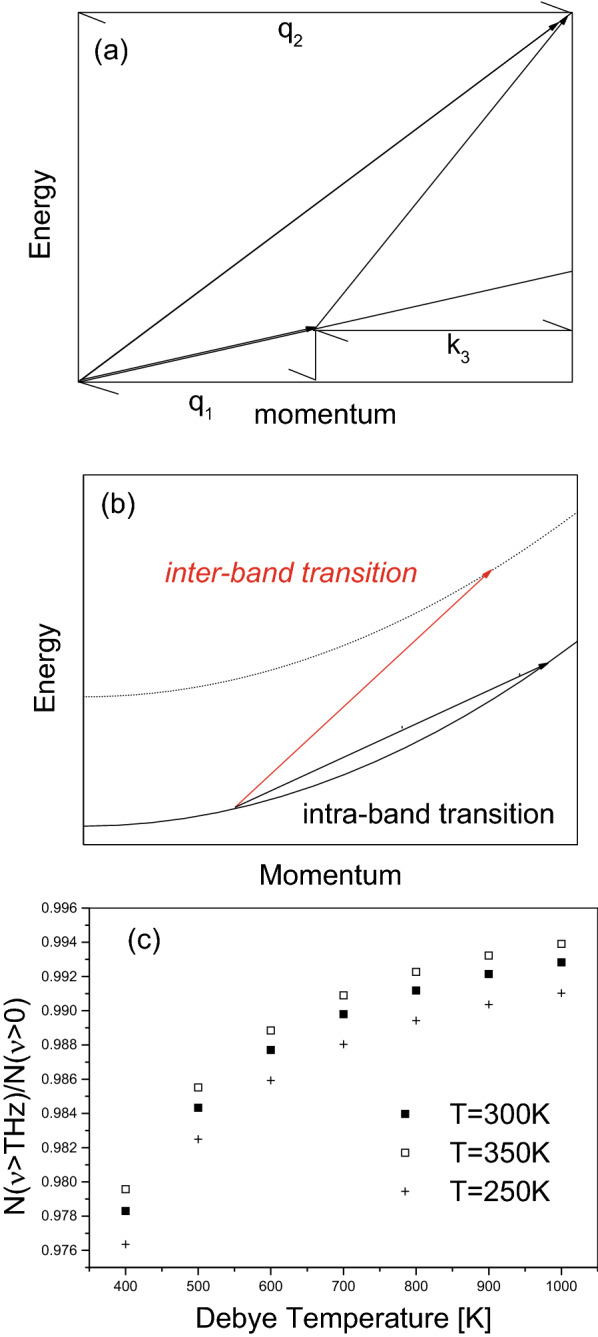
(**a**) A sketch illustrates that three waves with linear dispersion relation can form a closed energy–momentum conservation process. The slope of each straight-line segment represents its phase velocity. Because of $c_{tr}<c_{lo}<c$, $q_{2}=q_{1}+k_{3}$ and $c_{lo}q_{2}=\omega _{2}=\omega _{1}+E_{k}=c_{tr}q_{1}+ck_{3}$ can be satisfied, where $c_{lo}$ and $c_{tr}$ are phonon’s phase velocities and *c* is light velocity. (**b**) A sketch illustrates that a transition between two electronic states $k_{4},k_{5}$ within a same parabolic band cannot ensure the momentum difference $k_{5}-k_{4}=k_{3}$ and the energy difference $E_{5}-E_{4}=k_{5}^{2}-k_{4}^{2}$ to satisfy a linear dispersion relation $\frac{E_{5}-E_{4}}{k_{5}-k_{4}}=const$, which can be ensured by a transition between two states from different parabolic bands or $E_{5}=E_{4}+2k_{3}*k_{4}+k_{3}^{2}+E_{g}$. (**c**) The dependence of the ratio between the number of phonons whose frequencies $>1$ THz and total number of phonons on Debye temperature.

Many authors have taken into account phonon scattering in radiation generation from semiconductor^36,43,51^. For metal film held by dielectric “holder”, environment temperature can be felt by the film due to the connection between the “holder” and vacuum chamber wall. Because of the difference between $g^{tr}$ and $g^{lo}$, radiation generation is easy to involve in three levels: one from a state below the pseudogap and two above. An electron initially at a state below-pseudogap is scattered by a high-energy longitudinal-wave phonon to a state above-pseudogap and then is scattered by a low-energy transverse-wave phonon to another state above-pseudogap, and then return to the state below-pseudogap by emitting a photon. Because both phonon and photon have linear dispersion relation and their phase velocities satisfy $c_{tr}\equiv \frac{\omega ^{tr}}{q}<c_{lo}\equiv \frac{\omega ^{lo}}{q}<c$ (because of different values of elastic constants for longitudinal-wave and transverse-wave^53^). As illustrated in Fig. 6, this inequality relation $\frac{\omega ^{tr}}{q}<\frac{\omega ^{lo}}{q}<c$ can warrant a closed energy–momentum conservation process among phonon and photon. Moreover, two electronic states within a same parabolic band cannot ensure the difference between their momentum and that between their energy to satisfy a linear dispersion relation hold by photon, but two states from different parabolic bands can. These features well illustrate why the beating between longitudinal-wave phonon and transverse-wave one can allow an inter-band transition. Why this beating cannot cause a 3*D* Fermi sphere ES to generate photon can also be explained (because any two electronic states are in a same parabolic band $E_{k}\sim k^{2}$). Moreover, as previously mentioned, because different parabolic bands correspond to different 2*D* shaped-parts of 3*D* wavefunctions, losing orthogonality among 2*D* shaped-parts also favor the dipole matrix elements for the transition $\left( k_{z}+q,m\right) \rightarrow \left( k_{z},n\right)$ to be $\ne 0$. Namely, there are two prohibitions on the dipole radiation generation, one is the orthogonality of 3*D* wavefunctions and the other is parabolic dispersion relation of the 1*D* plane-wave part of 3*D* wavefunctions.

Because sound speed in many metals can be $10^{3\sim 4}$ m/s-level, if $c_{lo}=5\times 10^{3}$ m/s and $c_{tr}=3\times 10^{3}$ m/s, there will be $\frac{k_{3}}{q_{2}}=\frac{c_{lo}-c_{tr}}{c-c_{tr}}\sim 2\times 10^{-5}$ and hence yielding a photon $\hbar k_{3}c\sim$ meV will need a phonon $\hbar q_{2}c_{lo}\sim \frac{c-c_{tr}}{c_{lo}-c_{tr}}\frac{c_{lo}}{c}\,{\text {meV}} \sim 2.5$ meV , which can be supplied sufficiently for most metals in room-temperature environment 300 K.

Rate equations of radiation generation based on 3-level model read

42 $$\begin{aligned} d_{t}P_{3}= & {} P_{1}P_{3}*\sigma _{13}-P_{3}P_{2}*\sigma _{23}; \end{aligned}$$

43 $$\begin{aligned} d_{t}P_{2}= & {} P_{3}P_{2}*\sigma _{23}-A*P_{2}; \end{aligned}$$

44 $$\begin{aligned} d_{t}P_{1}= & {} -P_{1}P_{3}*\sigma _{13}. \end{aligned}$$

As shown in Eq. (28), the dependence of $M_{1,2}$ on phonon strength $A_{q}^{2}\sim g_{BE}\left( \omega _{q};T\right)$ determines $A_{k_{3}-k_{1}}^{2}<A_{k_{3}-k_{2}}^{2}$ (because $g_{BE}\left( \omega _{q_{13}};T\right) <g_{BE}\left( \omega _{q_{23}};T\right)$ if $q_{13}>q_{23}$), and hence $M_{1,3}<M_{2,3}$ or $\sigma _{13}<\sigma _{23}$.

As previously mentioned, eV-level gap between atomic levels can lead to $A\sim 10^{9}\,\text {s}^{-1}$^50^. Thus, even if assuming meV-level gap exists in atomic ES, corresponding *A* will be $\sim 10^{0}$ because of $A\sim \omega _{0}^{3}$ as shown in Eq. (26). Luckily, in the ES above-described, the $\xi$-value is $\sim 2\pi /\sqrt{k_{x}^{2}+k_{y}^{2}} \sim \upmu \text {m}$, about $10^{5}$-folds of that of atomic one. Thus, the *A*-value in the new ES can reach $\sim 10^{10}\,\text {s}^{-1}$. Moreover, for many metals with a Fermi sphere ES, phonon scattering can lead to a relaxation-time about $10^{-13}\sim 10^{-14}$ s or $\sigma \sim 10^{13}\sim 10^{14}\,\text {s}^{-1}$^45^. The new ES enables these $\sigma$-values to be kept at this level.

Actually, above-mentioned two characteristic time scales: 1/*A* and $1/\sigma$, represent respectively the strength of electron-photon interaction/coupleing and that of electron-phonon one, which are expectation values of two perturbation energies on electrons $\int \psi _{k+q}^{*}H_{e-photon}\psi _{k}d^{3}r$ and $\int \psi _{k+q}^{*}H_{e-phonon}\psi _{k}d^{3}r$, where $H_{e-photon}=eE\cdot r$ and $H_{e-phonon}=u\cdot \nabla V$. Here, under room temperature environment, ionic displacement *u* can be estimated, according to usual values of elastic constant *K* of metals $\sim 10^{2}\,\text {GPa}=10^{11}\,\text {J/m}^{3}$^44^ for yielding elastic energy $\frac{1}{2}Ka^{2}*u\sim 1\,\text {meV}=1.6\times 10^{-22}\,\text {J}$, where $a\sim 100pm$ represents the size of a cell, to be $\sim 10^{-2} a\sim 1pm$. Such a displacement, $10^{-2}$ folds of inter-atom distance *a*, can correspond to a $10\,\text {meV}$-level energy perturbation on an electron because the inner electric field *V*/*a* in most metals can be at *V*/[100*pm*]-level. Namely, for most metals in 300*K* environment, $\int \psi _{k+q}^{*}H_{e-photon}\psi _{k}d^{3}r<\int \psi _{k+q}^{*}H_{e-phonon}\psi _{k}d^{3}r$, especially $\int \psi _{k+q}^{*}H_{e-THz}\psi _{k}d^{3}r<<\int \psi _{k+q}^{*}H_{e-phonon}\psi _{k}d^{3}r$, can be warranted.

Note that the energy increment of an excited electron is not the kinetic energy of a phonon, instead, it is the variation of Coulomb energy associated with the ionic displacement, $u\cdot \nabla V_c$, that contribute this energy increment. Although the phonons number is not too large, electronic excitation is still very obvious. Therefore, it is not necessary to seek for applying large-amplitude THz accoustic wave on the metal or inputting large mount of phonons. This greatly lessen experiment condition requirement. Otherwise, we have to seek for a powerful THz mechanics vibration source whose difficulty is illustrated as follows: to input THz accoustic wave into the metal, we utilize the well-known formula reflecting cantilever transverse oscillation frequency $\nu$ inverse proportional to its extruded length *L* (see page 120–121 of ref.^53^), i.e. $\nu =\frac{\pi ^2}{L^2}\sqrt{\frac{EI}{\rho S}}$, for a typical metal material with following representative values: elastic module $E=2\times 10^{11}\,\text {Pa}=2\times 10^{11}\,\text {kg}\times \frac{1}{m}\times \frac{1}{s^2}$, cross-section area $S=1\times 10^{-6}\,\text {m}^2$, density $\rho =2700\,\text {kg/m}^3$, moment of inertia $I=ab^3/12$, $I/S\sim \,\text {m}^2$, extruding length $L=10^{-3}$ m,there will be $\nu \sim$ MHz. Namely, it is difficult to make an object whose material is common and size is in a macroscopic scale ($>\upmu {\text {m}}$) to do a vibration with a frequency at THz. A man-made macroscopically-sized THz mechanics vibration source is nearly not available.

But the metal’s “native” THz phonons are of sufficient mount at 300 K. Because the number of phonons at a specified frequency $\nu$ is the product of Bose–Einstein distribution function $g_{BE}$ at $\nu$ and phonon DOS at $\nu$: $n\left( \nu \right) =\frac{1}{\exp \left( \frac{\hbar \nu }{K_B T}\right) -1}*\nu ^2$, although low-energy phonon have very large $g_{BE}$-value, their low DOS-value $\nu ^2$ determine their number are near 0 at the $\nu \sim 0$ end. For most metals, their Debye temperature values $\Theta$, (or their highest phonon frequencies), are $200\sim 400$ K^44,45^, thus, the total phonon number $\int _0^{\Theta K_B/T}n\left( \nu \right) d\nu$ can have a large fraction $\int _{1THz}^{\Theta K_B/T}n\left( \nu \right) d\nu$ whose frequencies are $>1THz$, i.e. $\frac{\int _{1THz}^{\Theta K_B/T}n\left( \nu \right) d\nu }{\int _0^{\Theta K_B/T}n\left( \nu \right) d\nu }\gg 0$ if $T=300$ K and $\Theta =400$ K or higher. Fig. 6c indicates that this ratio is close enough to 1 at some typical situations. Moreover, the frequency corresponded by maximum phonon DOS in many metals can be up to tens meV^44,45^, therefore a metal’s “native” THz phonons are in sufficient mount for pumping electrons.

**Figure 7:**
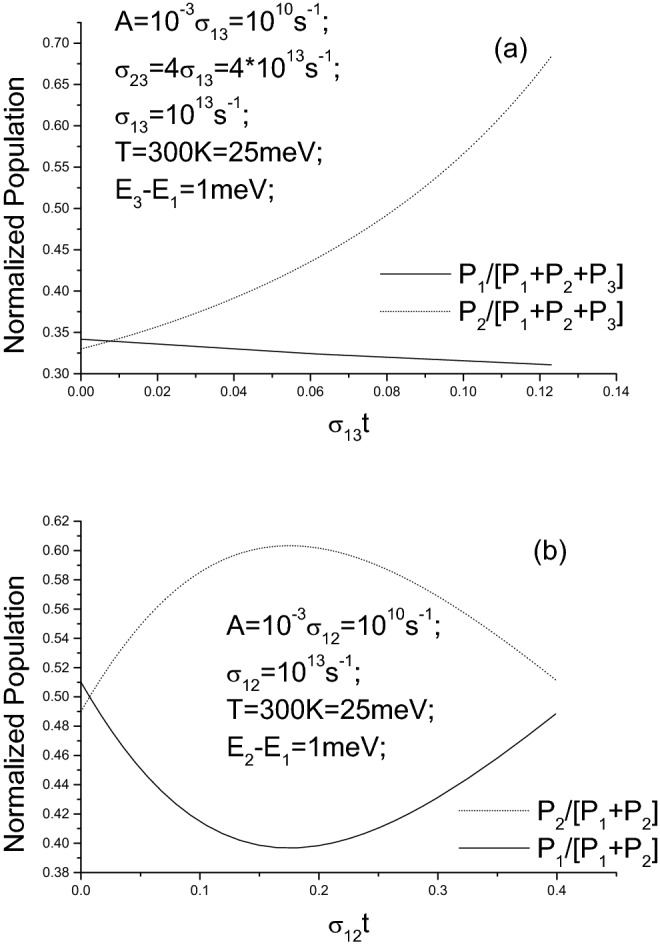
Examples of solutions of laser rate equations. (**a**) for 3-levels model and (**b**) for 2-levels model.

By choosing suitable values of controllable parameters, rate equations for 3-level model can yield solutions satisfying population inversion. This can be referred to Fig. 7. Actually, even for 2-level model, suitable values of controllable parameters can also warrant population inversion even for 2-level model (see Fig. 7).

The output power of a mono-color dipole oscillation $$e*d*sin\left( \omega t\right) $$ is $$P_{w}=\frac{e^{2}d^{2}\omega ^{4}}{12\pi \varepsilon _{0}c^{3}}=2.84\times 10^{-54}*[d]_{m}[\omega ]_{Hz} Watt$$, where $$\varepsilon _{0}=8.85\times 10^{-12}F/m$$, $$c=3\times 10^{8}m/s$$, $$e=1.6\times 10^{-19} C$$, dimensionless parameter $$[d]_{m}$$ is the value of *d* (dipole magnitude) in unit of meter, and $$[\omega ]_{Hz}$$ is defined likewise. Usually, free electron density in a simple metal is at $$10^{22\sim 23}\,{\text {cm}}^{-3}$$-level, which corresponds to an inter-atom interval at *nm*-level. Because THz frequency is non-transparent relative to metal medium with such a $$10^{22\sim 23}\,{\text {cm}}^{-3}$$-level density , it is only a thin layer near the surface that can have contribution to THz output. For a film $$1\,{\text {cm}}\times 1\,{\text {cm}}\times 0.01$$ cm, the thickness 0.01 cm is at the level of $$\lambda _{THz}=0.03$$ cm, the wavelength of THz photon, and hence it is suitable to view the layer of a 0.01 cm-thickness as the “skin” layer of a THz EM wave.

Thus, a metallic film $1\,{\text {cm}}\times 1\,{\text {cm}}\times 10^{-2}$ cm can contain $10^{20\sim 21}$ conduction-band electrons. For the new ES, states whose energies are within the range $\left[ E_{F}-0.05*K_{B}T,E_{F}+0.05*K_{B}T\right]$ have obvious contribution to various quantum transition processes, and the number of these states is estimated to be proportional to $0.1*K_{B}T/E_{F}$, which is $\sim 2.5\,{\text {meV}}/10\,\text {eV}\sim 10^{-4}$ for metals in room-temperature environment 300 K, and hence is $\sim 10^{16\sim 17}$. Of course, if the width of the range is $0.01*K_{B}T$, the number of these states will be $\sim 10^{15\sim 16}$, still higher several orders of magnitude than that of carriers in semiconductor. According to above formula, it is feasible to an output power at *Watt*-level from a film $1\,{\text {cm}}\times 1\,{\text {cm}}\times 10^{-2}$ cm which can contribute $10^{15}$ electrons to participate oscillation with a *THz* frequency and hence yield $10^{15}*\,{\text {meV}}=1.6\times 10^{-7}$ J energy over a time-scale at *ps*-level.

Moreover, it is easy to understand that intraband carrier acceleration by radiated THz electric field can lead to significant THz absorption and hence decrease the THz output power. Luckily, the THz-active layer is only of a thickness below the THz wavelength, $\sim 0.01\,{\text {cm}}<\lambda _{THz}$. Before $E_{x,y}$ is significantly depleted by intraband acceleration, it has transmitted the thin layer. Moreover, because space-asymmetric wavefunctions’ shape, intraband carrier acceleration to higher $k_x$-state might imply larger THz dipole moment and hence does not definitely imply “significant THz absorption”. A definite answer will be the task of future works.

Beside, the coupling loss to free-space THz radiation is also a factor affecting THz output power. For outstanding main purpose, this work avoids to consider various complicated secondary effects/mechanisms affecting THz output power. For example, intraband carrier acceleration above-mentioned can ”heat” carriers and then ”heat” ions, through carrier-ion collisions, and cause phonons energy rising and then in turn cause more THz EM generation. The main purpose is on the feasibility of fields-manipulating ES for achieving a specified wavelength EM output. These secondary effects/mechanisms will be content of future works.

## Summary

Fields-manipulating the ES of a metal enables high-density free electrons to form a new “volatile” ES, which can couple with acoustic phonon to achieve population inversion between states spaced by meV-level pseudogap. The fact that mobile electrons in metal is higher several orders of magnitude than that in semiconductor determines the technical scheme presented here to be of marked application prospect.

## Data Availability

All data generated or analysed during this study are included in this published article (and its Supplementary information files).

## Appendix

Detailed formulas are presented here. From Eqs. (10–12, 19, 20), we have

45 $$\begin{aligned}&\partial _{y}ln\phi =\frac{s+1}{2s}\partial _{u}ln\phi +\frac{s-1}{2s}\partial _{v}ln\phi ; \end{aligned}$$

46 $$\begin{aligned}&\partial _{x}ln\phi =\frac{s-1}{2s}\partial _{u}ln\phi -\frac{s+1}{2s}\partial _{v}ln\phi ; \end{aligned}$$

47 $$\begin{aligned}&\frac{1}{2}\partial _{u}\left[ N-M\right] -\frac{1}{2s}\partial _{u}\left[ N+M\right] -\frac{1}{2}\partial _{v}\left[ N+M\right] -\frac{1}{2s}\partial _{v}\left[ N-M\right] \nonumber \\&\quad =iws\frac{s+1}{s^{2}+1}u+iws\frac{s-1}{s^{2}+1}v, \end{aligned}$$

Substituting Eqs. (45, 46) back into Eqs. (19, 20), we have

48 $$\begin{aligned} \partial _{u}ln\phi= & {} s\frac{s+1}{s^{2}+1}\left[ N-iwxy\right] +s\frac{s-1}{s^{2}+1}M; \end{aligned}$$

49 $$\begin{aligned} \partial _{v}ln\phi= & {} -s\frac{s-1}{s^{2}+1}\left[ N-iwxy\right] +s\frac{s+1}{s^{2}+1}M; \end{aligned}$$

which “$$\Longrightarrow $$”

50 $$\begin{aligned} \partial _{v}\left\{ \frac{s+1}{s^{2}+1}\left[ N-iwxy\right] +\frac{s-1}{s^{2}+1}M\right\} =\partial _{u}\left\{ -\frac{s-1}{s^{2}+1}\left[ N-iwxy\right] +\frac{s+1}{s^{2}+1}M\right\} \end{aligned}$$

$$\Longrightarrow $$

51 $$\begin{aligned} \partial _{v}\left\{ \frac{s+1}{s^{2}+1}N+\frac{s-1}{s^{2}+1}M\right\} +\partial _{u}\left\{ \frac{s-1}{s^{2}+1}N-\frac{s+1}{s^{2}+1}M\right\} =iw\left[ \frac{s+1}{s^{2}+1}\partial _{v}\left( xy\right) +\frac{s-1}{s^{2}+1}\partial _{u}\left( xy\right) \right] ; \end{aligned}$$

$$\Longrightarrow $$

52 $$\begin{aligned}&s\partial _{v}\left( N+M\right) +\partial _{v}\left( N-M\right) +s\partial _{u}\left( N-M\right) -\partial _{u}\left( N+M\right) \nonumber \\&\quad =iw\{\left[ s+1\right] \left[ -\frac{\left( s^{2}-1\right) s^{2}}{\left( s^{2}+1\right) ^{2}}2v-\frac{2s^{3}}{\left( s^{2}+1\right) ^{2}}2u\right] +\left[ s-1\right] \left[ \frac{\left( s^{2}-1\right) s^{2}}{\left( s^{2}+1\right) ^{2}}2u-\frac{2s^{3}}{\left( s^{2}+1\right) ^{2}}2v\right] \}\nonumber \\&\quad =\frac{2iw}{\left( s^{2}+1\right) ^{2}} \left\{ -\left[ \left( s^{2}-1\right) s^{2}\left( s+1\right) +2s^{3}\left( s-1\right) \right] v +\left[ \left( s^{2}-1\right) s^{2}\left( s-1\right) -2s^{3}\left( s+1\right) \right] u\right\} \nonumber \\&\quad =\frac{2iw}{\left( s^{2}+1\right) ^{2}} \left\{ \left[ \left( 1-s\right) \left( s^{4}+4s^{3}+s^{2}\right) \right] v+\left[ \left( 1+s\right) \left( s^{4}-4s^{3}+s^{2}\right) \right] u\right\} \end{aligned}$$

From Eq. (47), its terms $\sim$ constant meet

53 $$\begin{aligned} \frac{1}{2}f_{1,0}-\frac{1}{2s}g_{1,0}-\frac{1}{2}g_{0,1}-\frac{1}{2s}f_{0,1}=0; \Longrightarrow \left[ sf_{1,0}-g_{1,0}\right] -\left[ sg_{0,1}+f_{0,1}\right] =0; \end{aligned}$$

its $\sim u^{1}$ terms meet

54 $$\begin{aligned}&\frac{1}{2}2f_{2,0}-\frac{1}{2s}2g_{2,0}-\frac{1}{2}g_{1,1}-\frac{1}{2s}f_{1,1}=iws\frac{s+1}{s^{2}+1}; \nonumber \\&\quad \Longrightarrow \left[ f_{2,0}-\frac{1}{s}g_{2,0}\right] -\left[ \frac{1}{2}g_{1,1}+\frac{1}{2s}f_{1,1}\right] =+iws\frac{s+1}{s^{2}+1}; \end{aligned}$$

its $\sim v^{1}$ terms meet

55 $$\begin{aligned}&\frac{1}{2}f_{1,1}-\frac{1}{2s}g_{1,1}-\frac{1}{2}2g_{0,2}-\frac{1}{2s}2f_{0,2}=iws\frac{s-1}{s^{2}+1}; \nonumber \\&\quad \Longrightarrow \left[ g_{0,2}+\frac{1}{s}f_{0,2}\right] -\left[ \frac{1}{2}f_{1,1}-\frac{1}{2s}g_{1,1}\right] =-iws\frac{s-1}{s^{2}+1}; \end{aligned}$$

Likewise, from Eq. (52), its terms $\sim$ constant meet

56 $$\begin{aligned} \left[ sg_{0,1}+f_{0,1}\right] +\left[ sf_{1,0}-g_{1,0}\right] =0; \end{aligned}$$

its $\sim u^{1}$ terms meet

57 $$\begin{aligned} \left[ sg_{1,1}+f_{1,1}\right] +\left[ s*2f_{2,0}-2g_{2,0}\right] =\frac{2iw}{\left( s^{2}+1\right) ^{2}}\left[ \left( s+1\right) \left( s^{4}-4s^{3}+s^{2}\right) \right] ; \end{aligned}$$

its $\sim v^{1}$ terms meet

58 $$\begin{aligned} \left[ s*2g_{0,2}+2f_{0,2}\right] +\left[ sf_{1,1}-g_{1,1}\right] =-\frac{2iw}{\left( s^{2}+1\right) ^{2}}\left[ \left( s-1\right) \left( s^{4}+4s^{3}+s^{2}\right) \right] ; \end{aligned}$$

For the dispersion relation Eq. (21), its $\sim u^{1}$ terms

59 $$\begin{aligned} f_{1,1}+f_{0,0}f_{1,0}+2g_{2,0}+g_{0,0}g_{1,0}=0; \end{aligned}$$

its $$\sim v^{1}$$ terms

60 $$\begin{aligned} g_{1,1}+g_{0,0}g_{0,1}+2f_{0,2}+f_{0,0}f_{0,1}=0; \end{aligned}$$

$$\partial _{x}Eq. (21)$$ and $$\partial _{y} Eq. (21)$$ read

61 $$\begin{aligned}&\frac{s-1}{2s}\left[ \partial _{uu}\left( N+M\right) +\left( N+M\right) \partial _{u}\left( N+M\right) \right] \nonumber \\&\quad -\frac{s+1}{2s}\left[ \partial _{vv}\left( N-M\right) +\left( N-M\right) \partial _{v}\left( N-M\right) \right] =0; \end{aligned}$$

62 $$\begin{aligned}&\frac{s+1}{2s}\left[ \partial _{uu}\left( N+M\right) +\left( N+M\right) \partial _{u}\left( N+M\right) \right] \nonumber \\&\quad -\frac{s-1}{2s}\left[ \partial _{vv}\left( N-M\right) +\left( N-M\right) \partial _{v}\left( N-M\right) \right] =0; \end{aligned}$$

which means

63 $$\begin{aligned}&\left[ \partial _{uu}\left( N+M\right) +\left( N+M\right) \partial _{u}\left( N+M\right) \right] =0; \end{aligned}$$

64 $$\begin{aligned}&\left[ \partial _{vv}\left( N-M\right) +\left( N-M\right) \partial _{v}\left( N-M\right) \right] =0; \end{aligned}$$

whose constant terms satisfies

65 $$\begin{aligned}&2g_{2,0}+g_{0,0}g_{1,0}=0; \end{aligned}$$

66 $$\begin{aligned}&2f_{0,2}+f_{0,0}f_{0,1}=0. \end{aligned}$$

Moreover, because of

67 $$\begin{aligned} 2ik_{x}=g_{0,0}-f_{0,0}; 2ik_{y}=g_{0,0}+f_{0,0}, \end{aligned}$$

there are

68 $$\begin{aligned} g_{0,0}=i\left( k_{y}+k_{x}\right) ; f_{0,0}=i\left( k_{y}-k_{x}\right) . \end{aligned}$$

12 equations and formulas, (10 equations in Eqs. (53–60, 65–66) and 2 formulas in Eq. (68)), enable us to determine electronic structure through following procedure. Equations (53, 56) lead to

69 $$\begin{aligned} \left[ sf_{1,0}-g_{1,0}\right] =0=\left[ sg_{0,1}+f_{0,1}\right] ; \end{aligned}$$

Equations (54, 57, 65) lead to

70 $$\begin{aligned}&\left[ f_{2,0}-\frac{1}{s}g_{2,0}\right] =\frac{iw}{2}\frac{s+1}{\left( s^{2}+1\right) ^{2}}\left[ s^{3}-4s^{2}+s+s^{3}+s\right] =\frac{iw}{2}\frac{s+1}{\left( s^{2}+1\right) ^{2}}\left[ 2s^{3}-4s^{2}+2s\right] ; \end{aligned}$$

71 $$\begin{aligned}&\left[ \frac{1}{2}g_{1,1}+\frac{1}{2s}f_{1,1}\right] =\frac{iw}{2}\frac{s+1}{\left( s^{2}+1\right) ^{2}}\left[ s^{3}-4s^{2}+s-s^{3}-s\right] =\frac{iw}{2}\frac{s+1}{\left( s^{2}+1\right) ^{2}}\left[ -4s^{2}\right] ; \end{aligned}$$

Equations (55, 58, 66) lead to

72 $$\begin{aligned}&\left[ g_{0,2}+\frac{1}{s}f_{0,2}\right] =-\frac{iw}{2}\frac{s-1}{\left( s^{2}+1\right) ^{2}}\left[ s^{3}+4s^{2}+s+s^{3}+s\right] =-\frac{iw}{2}\frac{s-1}{\left( s^{2}+1\right) ^{2}}\left[ 2s^{3}+4s^{2}+2s\right] ; \end{aligned}$$

73 $$\begin{aligned}&\left[ \frac{1}{2}f_{1,1}-\frac{1}{2s}g_{1,1}\right] =-\frac{iw}{2}\frac{s-1}{\left( s^{2}+1\right) ^{2}}\left[ s^{3}+4s^{2}+s-s^{3}-s\right] =-\frac{iw}{2}\frac{s-1}{\left( s^{2}+1\right) ^{2}}\left[ 4s^{2}\right] ; \end{aligned}$$

Equations (71, 73) yield

74 $$\begin{aligned} f_{1,1}= & {} \frac{iws^{2}}{\left[ s^{2}+1\right] ^{3}}\left\{ \left[ s+1\right] \left[ -4s\right] -\left[ s-1\right] \left[ 4s^{2}\right] \right\} \nonumber \\= & {} \frac{4iws^{2}}{\left[ s^{2}+1\right] ^{3}}\left\{ -s^{3}-s\right\} =-\frac{4iws^{3}}{\left[ s^{2}+1\right] ^{2}}; \end{aligned}$$

75 $$\begin{aligned} g_{1,1}= & {} \frac{iws^{2}}{\left[ s^{2}+1\right] ^{3}}\left\{ \left[ s+1\right] \left[ -4s^{2}\right] +\left[ s-1\right] \left[ 4s\right] \right\} \nonumber \\= & {} \frac{4iws^{2}}{\left[ s^{2}+1\right] ^{3}}\{-s^{3}-s\}=-\frac{4iws^{3}}{\left[ s^{2}+1\right] ^{2}}. \end{aligned}$$

Moreover, Equations (59, 60, 65, 66) lead to (the last “$=$” is due to Eq. (68))

76 $$\begin{aligned} f_{1,1}= & {} -f_{0,0}f_{1,0}=-i\left( k_{y}-k_{x}\right) f_{1,0}; \end{aligned}$$

77 $$\begin{aligned} g_{1,1}= & {} -g_{0,0}g_{0,1}=-i\left( k_{y}+k_{x}\right) g_{0,1}, \end{aligned}$$

Equations (69, 76, 77) yield

78 $$\begin{aligned} g_{1,0}= & {} sf_{1,0}=\frac{is}{k_{y}-k_{x}}f_{1,1}; \end{aligned}$$

79 $$\begin{aligned} f_{0,1}= & {} -sg_{0,1}=-\frac{is}{k_{y}+k_{x}}g_{1,1}. \end{aligned}$$

Substituting Eqs. (68, 78, 79) into Eq. (21), we have

80 $$\begin{aligned} E_{k}/\alpha= & {} k_{z}^{2}+g_{1,0}+\frac{1}{2}g_{0,0}^{2}+f_{0,1}+\frac{1}{2}f_{0,0}^{2} \nonumber \\= & {} k_{z}^{2}+k_{y}^{2}+k_{x}^{2}+\frac{is}{k_{y}-k_{x}}f_{1,1}-\frac{is}{k_{y}+k_{x}}g_{1,1}. \end{aligned}$$

Because *s* is an imaginary number, $f_{1,1}$ and $g_{1,1}$ are therefore complex-valued (see whether Eqs. (74, 75) contain terms $\sim s^{odd}$). Thus, $ImE_{k}=0$ will demand

81 $$\begin{aligned} 0=\frac{Imf_{1,1}}{k_{y}-k_{x}}-\frac{Img_{1,1}}{k_{y}+k_{x}}; \end{aligned}$$

$$\Longrightarrow $$

82 $$\begin{aligned} 0=\frac{0}{k_{y}-k_{x}}-\frac{0}{k_{y}+k_{x}}; \end{aligned}$$

The energy shift, or so-called “self-energy”, reads

83 $$\begin{aligned} ReE_{k}/\alpha -\left[ k_{z}^{2}+k_{\parallel }^{2}\right]= & {} \frac{is}{k_{y}-k_{x}}Ref_{1,1}-\frac{is}{k_{y}+k_{x}}Reg_{1,1} \nonumber \\= & {} \frac{4ws^{4}}{\left[ s^{2}+1\right] ^{2}}*\left[ \frac{1}{k_{y}-k_{x}}-\frac{1}{k_{y}+k_{x}}\right] =\frac{8ws^{4}}{\left[ s^{2}+1\right] ^{2}}*\left[ \frac{k_{x}}{k_{y}^{2}-k_{x}^{2}}\right] \nonumber \\= & {} \frac{8ws^{4}}{\left[ s^{2}+1\right] ^{2}}*\frac{1}{|k_{\parallel }|}*\frac{cos\left( \theta \right) }{cos\left( 2\theta \right) }. \end{aligned}$$

Note that because of $s^{2}<0$, the energy shift can be $\pm \infty$ if $s^{2}=-1$. According to the definition of *s* (see Eq. 9), it is feasible to achieve $s^{2}=-1$ under available values of those controllable parameters.
